# Mobile Cardiac Acoustic Monitoring System to Evaluate Left Ventricular Systolic Function in Pacemaker Patients

**DOI:** 10.3390/jcm11133862

**Published:** 2022-07-03

**Authors:** Jingjuan Huang, Weiwei Zhang, Changqing Pan, Shiwei Zhu, Robert Hardwin Mead, Ruogu Li, Ben He

**Affiliations:** 1 Department of Cardiology, Shanghai Chest Hospital, Shanghai Jiaotong University, Shanghai 200030, China; hjjxinhua@163.com (J.H.); zww1230523@163.com (W.Z.); pan-changqing@hotmail.com (C.P.); 18912834383@163.com (S.Z.); heben241@126.com (B.H.); 2 Silicon Valley Cardiology, East Palo Alto, CA 94303, USA; hardwin.mead@gmail.com

**Keywords:** mobile monitoring, acoustic cardiography, electromechanical activation time (EMAT), left ventricular systolic dysfunction (LVSD), left bundle branch pacing (LBBP)

## Abstract

The mobile cardiac acoustic monitoring system is a promising tool to enable detection and assist the diagnosis of left ventricular systolic dysfunction (LVSD). The objective of the study was to evaluate the diagnostic value of electromechanical activation time (EMAT), an important cardiac acoustic biomarker, in quantifying LVSD among left bundle branch pacing (LBBP) and right ventricular apical pacing (RVAP) patients using a mobile acoustic cardiography monitoring system. In this prospective single-center observational study, pacemaker-dependent patients were consecutively enrolled. EMAT, the time from the start of the pacing QRS wave to first heart sound (S1) peak; left ventricular systolic time (LVST), the time from S1 peak to S2 peak; and ECG were recorded simultaneously by the mobile cardiac acoustic monitoring system. LVEF was measured by echocardiography. A logistic regression model was applied to evaluate the association between EMAT and reduced EF (LVEF < 50%). A total of 105 pacemaker-dependent patients participated. The RVAP group (*n* = 58) displayed a significantly higher EMAT than the LBBP group (*n* = 47) (150.95 ± 19.46 vs. 108.23 ± 12.26 ms, *p* < 0.001). Pearson correlation analysis revealed a statistically significant negative correlation between EMAT and LVEF (*p* < 0.001). Survival analysis showed the sensitivity and specificity of detecting LVEF to be < 50% when EMAT ≥ 151 ms were 96.00% and 96.97% in the RVAP group. In LBBP patients, the sensitivity and specificity of using EMAT ≥ 110 ms as the cutoff value for the detection of LVEF < 50% were 75.00% and 100.00%. There was no significant difference in LVST with or without LVSD in the RVAP group (*p* = 0.823) and LBBP group (*p* = 0.086). Compared to LVST, EMAT was more helpful to identify LVSD in pacemaker-dependent patients. The cutoff point of EMAT for diagnosing LVEF < 50% differed regarding the pacing type. Therefore, the mobile cardiac acoustic monitoring system can be used to identify the progress of LVSD in pacemaker patients.

## 1. Introduction

Coronavirus disease 2019 (COVID-19) has swept nations across the world in the last three years, crippled health care systems, and caused a serious global pandemic. In-person clinic visits for regular follow-ups were delayed, and mobile monitoring in pacemaker patients become an important alternative and recommended by many medical societies. However, mobile monitoring activation usually requires programming steps during office access, transmitter registration, and patient consent. In addition, not all pacemakers have a mobile monitoring function. Visiting hospitals and clinics in person would consume limited medical resources and put patients at risk of infection.

Cardiac dysfunction has been demonstrated in a significant portion of patients with pacemakers, especially for high proportions of right ventricular pacing (VP) [1]. Long-term right ventricular apex pacing (RVAP) causes electrical and mechanical asynchrony, which can further lead to heart failure (HF), with decreased left ventricular ejection fraction (LVEF) and increased hospitalizations due to HF [2]. Left bundle branch pacing (LBBP) can circumvent the blocked site in the cardiac conduction system to produce near-physiological pacing for patients with bradycardia or HF [3]. The early identification of left ventricular systolic dysfunction (LVSD) is critical in managing pacing-induced HF and preventing unfavorable cardiovascular events [4]. 

Echocardiography is a mature tool to assess left ventricular systolic disfunction, but it can only be conducted in imaging centers, is not widely available to all ambulatory patients, and represents a short-term hemodynamic state during resting examination. The measurement of brain-type natriuretic peptide (BNP) also plays an important role in excluding acute decompensated HF. Nevertheless, the BNP level may be under the influence of many factors, such as renal function, age, and medicines, making interpretation complicated [5]. Neither echocardiography nor BNP level can be applied to outpatients for remote follow-up. It would be desirable to have a mobile and reliable method for pacemaker patients to evaluate LVEF anytime and anywhere, assisting the diagnosis of LVSD and providing early warnings about HF.

Acoustic cardiography is a technique that synchronizes heart sound with ECG and provides a comprehensive evaluation of the cardiac mechanical efficiency and electrical activity [6]. Electromechanical activation time (EMAT), as an important cardiac acoustic biomarker, measures the time interval from the onset of QRS to the peak first heart sound (S1). It represents the time required by the LV systole to produce sufficient pressure to close the mitral valve and is related to the acceleration of LV pressure. For patients without pacemakers, prolonged EMAT was significantly associated with LVSD, while shorter EMAT was associated with improved LV contractility and shortened electromechanical delay [7,8,9]. However, for patients with pacemakers, especially LBBP patients, whether EMAT can still be a parameter for detecting LVSD is unknown. To address this problem, Wenxin Tech. (Beijing, China) and Bayland Scientific (Pleasanton, CA, USA) developed a novel mobile acoustic monitoring system: a band-aid-like wearable electrocardiograph (ECG) and acoustic cardiography. By simply attaching the device to the chest, patients can perform ECG and PCG tests at home.

The objective of this study is to evaluate the diagnostic value of EMAT in LVSD in RVAP and LBBP patients by the mobile acoustic cardiography monitoring system.

## 2. Materials and Methods

### 2.1. Participants and Study Design

In this prospective study, pacemaker patients with VP dependency were consecutively enrolled at Shanghai Chest Hospital between April 2021 and October 2021. Patients were grouped by the VP types into RVAP or LBBP group. According to LVEF, each group was further divided into the LVSD subgroup and the non-LVSD subgroup. LVSD was defined as the presence of LVEF < 50% using echocardiography. Non-LVSD patients had LVEF ≥ 50% and no HF-related clinical manifestations. The demographic and baseline clinical data of all subjects were recorded. Informed consent was obtained from each participant, and the study protocol was approved by the ethics committee of the hospital. The study was compliant to the principles outlined in the Declaration of Helsinki.

### 2.2. Inclusion and Exclusion Criteria

Inclusion criteria were pacemaker patients with VP dependency, implantation of dual-chamber pacemaker for second- or third-degree atrioventricular block, single-chamber pacemaker for atrial fibrillation with slow ventricular rate, and cardiac resynchronization therapy (CRT) for HF with complete left bundle branch block (CLBBB). VP dependency was defined as a daily VP proportion ≥ 90% during interrogation. 

Exclusion criteria included age < 18 years old, unstable arrhythmias, such as paroxysmal atrial fibrillation, and frequent ventricular tachycardia. We also excluded patients with severe chronic obstructive pulmonary diseases, uncontrolled hypertension, severe valvular heart diseases, end-stage renal failure, constrictive pericarditis, and psychological problems. 

### 2.3. Mobile Cardiac Acoustic Monitoring System

**Figure 1:**
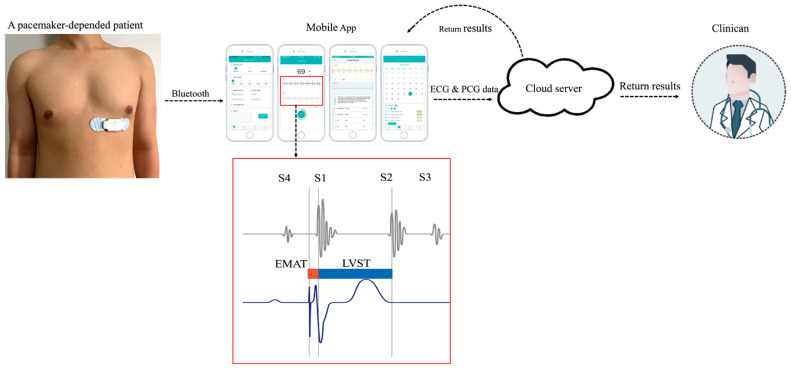
Illustration of the WENXIN^®^ device on patient with pacemaker. The typical relationships between ECG and cardio-hemic vibrations recorded are summarized.

WENXIN^®^ device (Wenxin Tech. and Bayland Scientific, Beijing, China and Pleasanton, CA, USA) was used to record heart sound and ECG data in each participant. All participants were placed in supine position. The device consists of two parts, a reusable centerpiece with an embedded sound sensor and a disposable patch with two electrodes. When in use, click the patch onto the centerpiece and connect it to the patient’s chest on the V5 standard precordial position. The recording device has an embedded sound sensor in the middle and two electrodes on each side. The electrodes create a single-lead ECG signal while the sound sensor collects the data of the heart sounds. The device is powered by a rechargeable lithium battery. The other details have been described previously [10]. Digital data can be collected by connecting the device to a smartphone or tablet app via Bluetooth and then the data being sent to a cloud-based data center for analysis and archiving. An automatic analysis software designed and developed by Wenxin Tech. was applied to real-time annotation. The following cardiac acoustic parameters related to LV systolic function were assessed separately or in combination, and each parameter was measured three times and its average value was used in annotation. The analysis results could be returned to mobile app and clinicians (Figure 1).

(1) EMAT: the time interval from the start of the pacing ECG Q wave to S1 peak.
(2) EMAT%: the ratio of EMAT to the RR interval, which is the proportion of the cardiac cycle occupied by EMAT.
(3) Left ventricular systolic time (LVST): the time from S1 peak to S2 peak.
(4) LVST%: the ratio of LVST to the RR interval.

### 2.4. Echocardiography

Transthoracic echocardiographic data were obtained by the GE Vivid system (Vivid E9 or Vivid E95, GE Medical Systems, Horten, Norway). LV end-diastolic volumes and LV end-systolic volumes were obtained from apical four- and two-chamber views. The modified biplane Simpson’s rule was used to measure LVEF. The average of three measurements was used for the analysis. The echocardiographer was blinded to all other clinical data and acoustic cardiography findings.

### 2.5. Standard 12 Lead ECG Measurements and Interval Definitions

QRS duration was the interval corresponding to the longest QRS duration measured by a standard 12-lead simultaneous body surface ECG. QR-interval was measured from QRS onset at the earliest deflection to the peak of the R wave (or S wave for QS pattern) in lead V5 [11]. According to the paced QRS axis, patients were divided into three different types as follows: axis between 0° and 90° (a normal axis), axis between 0° and −90° (left axis deviation), and axis within 90° and 120° (right axis deviation) [12]. All ECGs and endocardial measurements were displayed at a paper speed of 50 mm/s. Three continuous QRS complexes were measured by two independent and experienced ECG specialists, and the averaged values were recorded. 

### 2.6. Statistical Analysis

Descriptive statistics include the frequencies and percentages for categorical data and the mean ± standard deviation (SD) for continuous data. Descriptive statistics were conducted for all baseline characteristics, stratified by RVAP or LBBP group. We applied *t*-test for continuous variables and chi-square analysis for categorical variable among two groups. Pearson correlation analysis was used to test the correlation between EMAT and LVEF. To evaluate EMAT as a predictor of LVSD, multivariate logistic regression analysis with correction for contextual measurements was used. Receiver operating characteristic (ROC) curve analysis was applied to select the optimal EMAT cutoff value that best differentiates LVSD patients from non-LVSD patients in RVAP or LBBP group. SPSS software Version 22.0 (SPSS, Inc., Chicago, IL, USA) was used for statistical analysis. A two-tailed *p* value < 0.05 was considered statistically significant.

## 3. Results

### 3.1. Comparison of Baseline Clinical Characteristics, ECG Pattern, and Cardiac Acoustic Biomarkers of Patients with RVAP and LBBP

A total of 105 adult patients were identified and included in the study. All subjects were divided into two groups according to whether the lead was positioned on the conventional right ventricular apex (*n* = 58) or at the left bundle branch area (*n* = 47). The mean age was 69.52 ± 12.11 years, and 64.76% of the patients were male. Twenty-one patients in the LBBP group were implanted with CRT due to HF combined with CLBBB. These two groups were clinically similar except for the paced QRS characteristics and cardiac acoustic biomarkers. The QRS duration was longer in the RVAP group (172.60 ± 35.48 ms) than in the LBBP group (145.32 ± 34.48 ms; *p* < 0.001). The EMAT was obviously higher in the RVAP group than in the LBBP group (150.95 ± 19.46 vs.108.23 ± 12.26 ms, *p* < 0.001). There was no significant difference between the RVAP group and LBBP groups in LVST (309.41 ± 79.83 vs. 312.34 ± 30.00, *p* = 0.812). Patients’ baseline demographic characteristics, ECG pattern, and cardiac acoustic biomarkers are shown in Table 1.

### 3.2. Correlation between LVEF and Cardiac Acoustic Biomarkers

**Figure 2:**
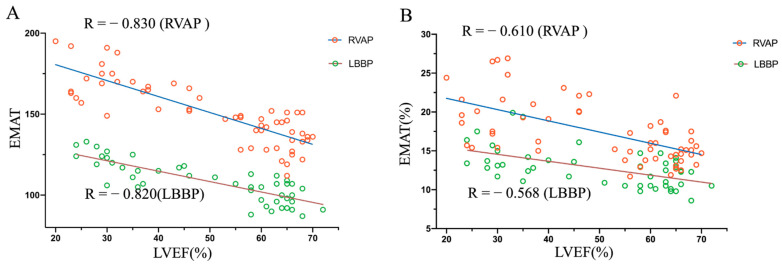
Correlation between LVEF and EMAT and EMAT% in pacemaker patients with RVAP and LBBP. (**A**) EMAT vs. LVEF in the patients with pacemaker. Red circles = RVAP (R = − 0.830; *p* < 0.001); green circles = LBBP (R = − 0.820; *p* < 0.001). (**B**) EMAT% vs. LVEF in the patients with pacemaker. Red circles = RVAP (R = − 0.610; *p* < 0.001); green circles = LBBP (R = − 0.568; *p* < 0.001).

In the RVAP group, EMAT vs. LVEF correlation coefficient = −0.830; EMAT% vs. LVEF correlation coefficient = −0.610 (both *p* < 0.001) (Figure 2). In the LBBP group, EMAT vs. LVEF correlation coefficient = −0.820; EMAT% vs. LVEF correlation coefficient = −0.568 (both *p* < 0.001) (Figure 2). From the Pearson correlation analysis, EMAT showed stronger correlation with LVEF compared with EMAT% in both RVAP and LBBP groups, suggesting that EMAT correlated best with the left ventricular systolic function.

**Figure 3:**
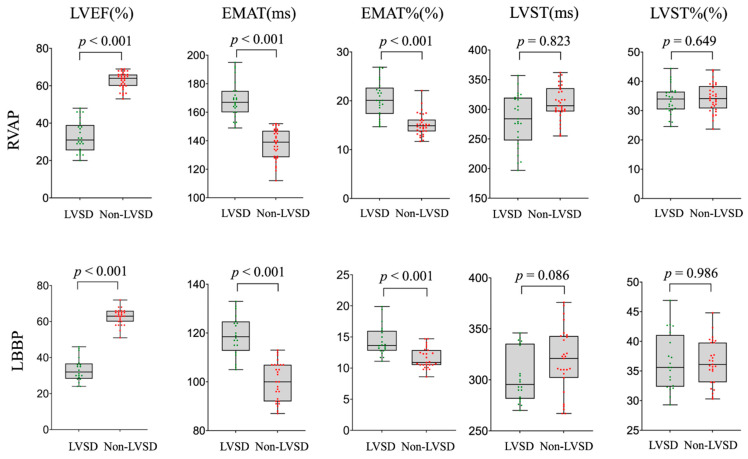
Comparison of cardiac acoustic biomarkers of patients with and without LVSD in RVAP and LBBP groups.

**Figure 4:**
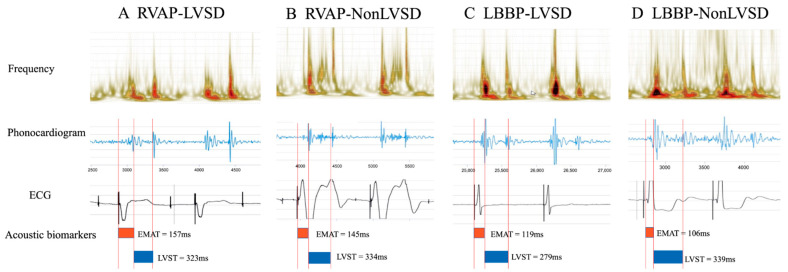
Examples of cardiac acoustic and ECG waveforms and automatic analysis results in different groups.

A comparison of cardiac acoustic biomarkers was conducted between LVSD and non-LVSD in RVAP and LBBP groups. The LVSD subgroup showed a significantly longer EMAT and EMAT% than the non-LVSD subgroup. Specifically, the EMAT was notably longer in the LVSD patients than in the non-LVSD subgroup in both RVAP and LBBP groups. However, a statistically significant difference was not discovered in LVST and LVST% between LVSD and non-LVSD subgroups (Figure 3 and Figure 4).

### 3.3. Performance of Cardiac Acoustic Biomarkers (EMAT Cutoff)

**Figure 5:**
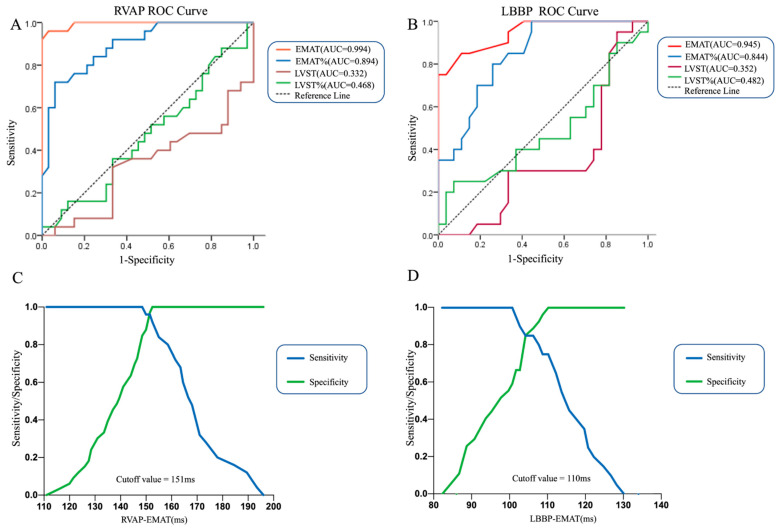
The diagnosis value of different cardiac acoustic biomarkers in LVSD. (**A**,**B**) Comparison of EMAT, EMAT%, LVST and LVST % ROC analysis to identify LVSD in RVAP and LBBP groups. (**C**,**D**) ROC analysis of EMAT for LVSD in RVAP and LBBP groups.

To evaluate the value of EMAT in the detection of LVSD among RVAP and LBBP patients, we first applied logistic regression using LVSD as the outcome and EMAT as the only predictor, and we repeated it using several other variables as single predictors as well, including EMAT%, LVST, and LVST%, respectively. Figure 5 displays the area under the curve (AUC) comparing the abilities of EMAT and EMAT% to identify LVSD in RVAP and LBBP groups. In both groups, EMAT is a better predictor than EMAT%. No statistical difference was observed between LVST and LVST%. We then performed a ROC analysis to determine the cutoff value of EMAT with optimal sensitivity and specificity. In the RVAP group, EMAT predicted LVSD with the sensitivity and specificity of 96.00% and 96.97%, respectively, for the best-selected cutoff (151 ms) (Figure 5C). In the LBBP group, EMAT identified LVSD with the best-combined sensitivity and specificity of 75.00% and 100.00%, respectively, at the cutoff value of 110 ms (Figure 5D). Therefore, we concluded that the RVAP group and LBBP group have different EMAT cutoff values.

## 4. Discussion

As far as we know, the study is the first publication analyzing LVSD in patients with pacemakers using the mobile cardiac acoustic monitoring system, especially in LBBP patients. Our results show that EMAT measurement is a fast and alternative diagnostic method with high accuracy for LV systolic dysfunction in pacemaker patients with VP dependency. We further determine cutoff values of EMAT in patients with RVAP and LBBP, respectively.

### 4.1. The Mobile Cardiac Acoustic Monitoring System Is Convenient, Especially Suitable for Contact-Less Monitoring during COVID-19

The benefits of adopting acoustic cardiography in pacemaker patients include relatively low costs, noninvasiveness, ease of use, and early recognition of LVSD [13]. Unlike conventional diagnostic methods that require in-person assessment, the cardiac acoustic system we test has a mobile monitoring function that can analyze the data through the cloud environment and provide near-real-time results to doctors and patients. Using a reliable and accurate mobile system to monitor patient heart function state and changes can reduce unnecessary clinic visits, thus reducing the risk of transmission of COVID-19 in clinics. If repeated on a large scale of study, this device enables a safer evaluation of LV function than traditional in-person examination methods. For its mobility and ease of use, this technology of detecting LVSD can be performed regularly at home as a trigger for further interventions rather than obtaining echocardiograms at regular intervals, contributing to improved medical resource utilization.

### 4.2. EMAT Was Reliable and Effective in Assisting the Diagnosis of LVSD in Pacemaker Patients

EMAT reflects the LV isovolumic contraction phase and is the time required for ventricular contraction to close the mitral valve. It has been shown that EMAT is associated with pulsed-wave Doppler echocardiographic parameters of aortic outflow or mitral inflow in CRT patients with stable HF [14]. EMAT and related parameters have been used to effectively guide the medical treatment of HF and improve clinical outcomes in patients [15]. Previously, Zuber et al. demonstrated EMAT strongly agreed with echocardiographic LVEF measured value to distinguish LV systolic dysfunction in 161 HF patients [16]. Compared with BNP, EMAT predicted LVEF depression more accurately [16]. Roos M. et al. examined 37 HF patients who underwent cardiac catheterization by acoustic cardiography and found that EMAT was negatively correlated with LV dP/dt (r = −0.961, *p* = 0.063) in LVSD patients [17]. EMAT ≥ 104 ms has been determined as the cutoff value for the diagnosis of LVEF < 50%, resulting in high sensitivity and specificity in patients without pacemakers [10]. Dillier R. et al. considered an EMAT value more than 120 ms to be abnormally prolonged. They found that patients with acute and chronic HF had an average EMAT value of 122.0 ± 29.4 ms and 118.0 ± 24.3 ms, respectively, while the average EMAT value of volunteers with normal heart function was 89.7 ± 16.1 ms [18]. Not only that, Michel et al. concluded that the reproducibility of EMAT was the highest in 43 CRT patients, and the intra-observer variability in EMAT and echocardiography measurements were similar (9.9% vs. 8.5%) [14]. 

Consistent with the above findings, we found that the LVSD subgroup had significantly prolonged EMAT, which coincided with a decreased LVEF, compared with the non-LVSD subgroup in RVAP (168.80 ± 12.74 ms vs. 137.40 ± 10.73 ms, *p* < 0.001) and LBBP groups (119.00 ± 8.24 ms vs. 100.30 ± 7.92 ms, *p* < 0.001). The Pearson correlation analysis indicated that EMAT and LVEF were significantly negatively correlated. Although more patients received CRT for HF and CLBBB in the LBBP group than the RVAP group, the LVEF of many LBBP patients greatly improved with the extension of implantation time. Therefore, there was no significant difference in LVEF between the RVAP group and the LBBP group during the follow-up of phonocardiogram. All these findings show the reliability and validity of EMAT in assisting the diagnosis of LVSD in patients with pacemakers.

### 4.3. Different Cutoff Values of EMAT between RVAP and LBBP

Normal cardiac conduction begins in the sinoatrial node and then spreads through the His–Purkinje network and generates a narrow QRS complex with all regions of the LV electro-mechanical activation within a short time. On the contrary, by directly pacing the myocardium without utilizing the His–Purkinje system, cardiomyocytes are activated cell by cell. Therefore, RV pacing produces a non-physiological activation sequence. RVAP patients with wider QRS duration and iatrogenic LBBB were at high risk of pacing-induced cardiomyopathy [19]. RVAP also prolongs mitral regurgitation by increasing pre-ejection and relaxation times, resulting in prolonged S1 in mitral valve closure [20]. 

Compared to conventional RVAP, LBBP could produce narrower-paced QRS waves and become a novel physiologic pacing method. Some previous studies have proven the stability and feasibility of LBBP, including fewer complications, while also being easier to implant [21,22]. LBBP is considered an alternative approach for conduction system pacing that can cross the blockage and ensure the electrical synchronization of the LV at levels comparable to intrinsic LV activation [20]. The characteristics of LBBP include short QRS duration, high R wave, low pacing threshold, rapid synchronous activation of the left ventricle, and correction of left bundle branch block. Consequently, LBBP may be a feasible choice for a high burden of RVAP patients or CRT candidates.

Since RVAP and LBBP have different cardiac conduction times and synchronization, in this study, we analyzed the data and determined the EMAT cutoff value for each group. The results show that the cutoff value of EMAT in the RVAP group was remarkably longer than that in the LBBP group. We found that the sensitivity and specificity of using EMAT ≥151 ms as the cutoff value for the detection of LVEF < 50% were 96.00% and 96.97% in patients with RVAP. Meanwhile, the sensitivity and specificity of using EMAT ≥110 ms as the cutoff value for the detection of LVEF < 50% were 75.00% and 100.00% in patients with LBBP.

### 4.4. Roles of Other Cardiac Acoustic Biomarkers in the Diagnosis of LVSD

Some researchers believe that it is necessary to analyze the proportion of EMAT in the cardiac cycle (EMAT%) because EMAT can be influenced by heart rate difference among individuals induced by neurohumoral factors [17]. Efstratiadis et al. found that HF patients with prolonged EMAT% had decreased ventricular systolic synchrony, increased LV systolic pressure, and decreased maximum LV dP/dt [9]. In addition, in their study, EMAT% ≥ 15% was a criterion of detection of LVSD, and EMAT% < 10%was 100% accurate in excluding LVSD [9]. Kamran et al. concluded that EMAT is unrelated to heart rate, and they argued against the necessity of correcting EMAT for heart rate based on animal experiments [23]. On the other hand, most of our patients’ heart rates are in the range of 60–80 beats per minute, and the fluctuation range is relatively small. There was no statistically significant difference in EMAT% between the LVSD subgroup and the non-LVSD subgroup in our study. Nevertheless, this may be related to the small sample size of this research. Future studies are needed to confirm this conclusion. 

The increase in the LVST interval is related to the decrease in systolic function, the increase in LV ejection time, and the prolongation of the systole. LVST% indicates the proportion of systole (pump function) versus diastole (filling) in the cardiac cycle [7]. Dillier et al. found that LVST showed no statistical significance in different monitoring periods or ages in asymptomatic patients. There was a significant difference in the LVST between the HF and normal group [13]. In contrast, we found no statistical significance in LVST and LVST% between the LVSD and non-LVSD subgroups. The discrepancies between our results and those of Dillier et al. may be due to different sample sizes, races, and other statistical factors.

### 4.5. Limitations

There are several limitations in our study. First, the data quality could be affected by exogenous and endogenous noise, including background and breathing noise. It is required to have the patient remain still and quiet during recording. Second, the study participants are limited to hospitalized patients with pacemakers. Future studies should consider examining the value of EMAT in the prognosis of HF in pacemaker patients over the long-term. Third, the number of patients in this study was relatively small. A larger sample size is needed to establish accurate cutoff values for each parameter in the future. Finally, we did not further analyze the predictive value of EMATs in the HF with preserved EF (HFpEF) population [24].

## 5. Conclusions

In conclusion, we demonstrated that EMAT value was associated with LVSD among pacemaker patients with different cutoff points in the RVAP and LBBP patients. The mobile cardiac acoustic monitoring system has great potential to be a fast, cost-effective, and contact-less way to assist detecting LVSD in patients with pacemakers, and it holds promise in home-monitoring of patients with HF if the findings are duplicated in a large-scale study. Further studies are needed to explore the application of acoustic cardiography in pacemaker patients in predicting cardiac outcomes.

## Figures and Tables

**Table 1:** Baseline patient characteristics according to baseline clinical, ECG pattern, and cardiac acoustic biomarkers.

	RVAP (*n* = 58)	LBBP (*n* = 47)	*p*
Male (*n* (%))	34(58.62)	34(72.34)	0.143
Age (years)	72.0 ± 12.45	66.47 ± 11.05	0.019
Indications for implantation			<0.001
AVB	32	13	
AF with slow ventricular rate	26	13	
HFrEF with CLBBB	0	21	
Pacemaker mode			<0.001
Dual chamber pacemaker	30	13	
Single chamber pacemaker	28	13	
CRT	0	21	
Days after implantation	89.83 ± 20.38	86.91 ± 27.13	0.268
Heart rate (bpm)	68.45 ± 15.45	71.68 ± 14.53	0.276
MAP (mmHg)	91.03 ± 13.17	90.48 ± 9.71	0.811
Paced QRSd (ms)	172.60 ± 35.48	145.32 ± 34.48	<0.001
QTc (ms)	498.97 ± 70.49	475.60 ± 48.74	0.056
QR interval in V5 (ms)	53.45 ± 18.43	46.28 ± 17.52	0.045
QRS axis, *n* (%)			<0.001
Normal	11	20	
Left axis deviation	33	18	
Right axis deviation	12	9	
LVEF (%) at follow-up	50.09 ± 16.39	50.21 ± 15.63	0.968
NYHA class, *n* (%)			0.302
II	32	24	
III	22	12	
IV	4	1	
EMAT (ms)	150.95 ± 19.46	108.23 ± 12.26	<0.001
EMAT% (%)	17.41 ± 3.90	12.53 ± 3.05	<0.001
LVST (ms)	309.41 ± 79.83	312.34 ± 30.00	0.812
LVST% (%)	34.05 ± 4.82	36.48 ± 4.18	0.008

Values are given as mean ± SD or *n* unless otherwise indicated. RVAP—right ventricular apical pacing. LBBP—left bundle branch pacing. AVB—atrioventricular block. AF—atrial fibrillation. HFrEF—heart failure with reduced ejection fraction. CLBBB—complete left bundle branch block. CRT—cardiac resynchronization therapy. MAP—mean arterial pressure. QRSd—QRS duration. QTc—corrected QT interval. LVEF—left ventricular ejection fraction. NYHA—New York Heart Association. EMAT— electromechanical activation time. LVST—left ventricular systolic time.

## Data Availability

The datasets generated and analyzed during the present study are available from the corresponding author on reasonable request.
